# The impact of marketing strategies in healthcare systems

**DOI:** 10.25122/jml-2019-1003

**Published:** 2019

**Authors:** Victor Lorin Purcarea

The dynamic evolution of life has inevitably affected the healthcare systems generating significant changes and imposing healthcare marketing as an indispensable element of health brands. Healthcare is a field in a permanent evolution, the plethora of opportunities stimulating creativity, enthusiasm, and will exploit the specialists in the field.

As the philosophy and marketing techniques in other fields cannot find applicability in the healthcare services, healthcare need their own approach and present certain features that are not found in other industries (Thomas RK. Health Services Marketing, A Practitioner’s Guide, 2008, Ed. Springer).

Through its specificity, healthcare marketing is an interdisciplinary field because it uses certain concepts, methods, and techniques specific both to classical and social marketing. The specificity of healthcare marketing is that there are services and markets but no money equivalent. This means the effectiveness of its application can be found in the image of a healthy population, the detection of a chronically ill category of people, ensuring the treatment of ill people by going through the whole rehabilitation process, professional reintegration, social reintegration of ill people, etc. The application of marketing in the field of healthcare was imposed by the problems in the health of the society.

An effective marketing approach involves in-depth investigation of the patients’ needs, identifying latent needs and offering new health services that patients have not explicitly requested (Purcarea VL. Marketingul Ingrijirilor de Sanatate - curs Universitar, 2017, Ed. Universitara “Carol Davila”).

Patients’ involvement in the achievement of the medical act has become a necessity of present life with wide and complex meanings, not only beyond changing the mentality of the providers but also with significant changes like lifestyle, consumption habits and medication of beneficiaries. As the daily process evolves, change will be fundamental to the fundamental purpose of our existence: life. In addition, this will undoubtedly bear the hindrance of how the relationship will harmonize the need for health. Structural changes forces health systems to accelerate towards the future, considering the current needs, and the future strategy cannot be viable without performing management and marketing abilities (Popa F, Purcarea Th, Purcarea VL, Ratiu M. Marketingul serviciilor de ingrijire a sanatatii, 2007, Ed. Universitara “Carol Davila”).

The marketing of healthcare services differs primarily through the nature of demand for health services. Secondly, the beneficiary may not be the target of the marketing campaign, the physician being the one who decides what, where, when, and how much will be provided for a particular service. The decision-maker may be the doctor, the health plan representative, a family member. Healthcare services also differ where the product can be very complex and may not be easily conceptualized. Many of the procedures used in healthcare, especially those based on technology, are complicated and difficult to explain to a person who is not specialized in that particular field.

Another healthcare challenge, especially for service providers, is that not all potential clients are considered “desirable” for a particular service. While service providers are required to provide services to all applicants, regardless of their ability to pay, there are certain categories of patients whom the marketer may not encourage to request a particular service. The marketer faces the challenge of attracting customers to healthcare organizations, however, without attracting too many from the category of those who are likely to represent economic debts.

Over the past decade, healthcare has experienced many marketing trends that have fundamentally altered marketing. These trends are the follows:

— From a mass marketing approach to a more specific approach.
— From image marketing to service marketing.
— From “one measure for all” to personalization.
— From the emphasis on a health episode to a long-lasting relationship.
— From “ignoring” the market, to market intelligence.
— From low-tech to high-tech (Thomas RK. Health Services Marketing, A Practitioner’s Guide, 2008, Ed. Springer).

Marketing plays an important role in helping healthcare professionals to create, communicate, and provide value to their target market. Modern marketers start from customers rather than from products or services. They are more interested in building a sustainable relationship, than in ensuring a single transaction. Their aim is to create a high level of consumer satisfaction so that they return to the same supplier. Marketers have used many traditional methods that include marketing research, product design, distribution, pricing, advertising, promotional sales, and sales management. These methods need to be joined by new ones, related to new technology and new concepts, to attract customers through messages and offers.

Although the consumer typically receives *most* of the information about a product through the commercial media. The most *important* information comes from recommendations or from publicly available independent authorities. The two categories of sources provide complementary functions; commercial media – informs, while personal or expert sources legitimize or potentiate the evaluation process. For example, physicians often find out about new drugs from commercial sources, but they turn to other physicians for legitimate opinions.

Although much has been written about subliminal decisions, current models look at the process from a cognitive perspective, which means the consumer/patient, makes his own judgements on a rational basis (Kotler P, Shalowitz J, Stevens RJ. Strartegic Marketing for Health Care Organizations. Building a Customer Driven Health System, 2008, Ed. Jossey-Bass, A Wiley Imprint).

In an attempt to meet a need, the patient expects certain benefits from the chosen health service and provider. The patients’ attitudes, judgements, and preferences about certain brands through a procedure of evaluating the attributes of these brands, develop a set of beliefs about the attributes that correspond to each brand (Kotler P, Rackham N, Krishnaswamy S. Ending the War between Sales and Marketing, July 2006, Harvard Business Review).

The main actor who marks the process of production and delivery of healthcare services is the patient and his presence *is part of the delivery system*.

In the field of healthcare services, the performance occurs only in the presence of the healthcare consumer and his wiliness to the service. Thus, the consumer becomes an indispensable factor for any service; the consumer interacts with the supplier, becomes co-provider of the service, participating with time and effort in the delivery process (Popa F, Purcarea Th, Purcarea VL. Ratiu M. Marketingul serviciilor de ingrijire a sanatatii, 2007, Ed. Universitara “Carol Davila”).

Healthcare consumers are actively or passively involved in delivery, but their presence has implications for the medical organization’s activity because any specialist who meets the patient will contribute to the service production. In addition, any tangible element, which the healthcare consumer meets, is part of the health service delivery process; in fact, any changes occurring at the place of service delivery, will lead to changes in the patient’s behavior.

From a marketing perspective, the process of providing healthcare services must be conducted in full compliance with patient requirements, activities are being designed to meet these requirements. However, achieving such a goal implies the identification of all points of interference of healthcare staff with the healthcare service consumers and the assessment of the extent to which the activities carried out at these points correspond to the needs and expectations expressed by the patients. Since the behavior of the health services consumer is difficult to predict, the presence of the patient in the delivery process may be a source of major uncertainty.

Far from having a passive behavior, the consumer has multiple functions in the production of services as a “co-producer”, which determines many specialists to consider him an “*external human resource*”.

Patient satisfaction must be the main objective of any healthcare organization and this requires a thorough knowledge of their needs and expectations. Providing a high-quality healthcare service is based on meeting certain requirements so that the service attains the level desired by the patient. In order to gain the trust of healthcare consumers, the specialized staff of the organizations in the field must be more receptive to the wishes, suggestions, patient complaints and, at the same time, become more sensitive to their concerns. The effectiveness of this approach depends on how the medical organization has an effective communication with patients, presents a correct image of the health service, and delivers the promised service properly, and presents a permanent concern for the continuous improvement of the service provided to exceed the expectations of the patients (Ciurea AV, Cooper CL, Avram E. (coord). Managementul sistemelor şi organizaţiilor sănătăţii, 2010, Editura Universitară “Carol Davila”, Bucureşti).

By acting in a dynamic and unpredictable environment, (in order to survive) the health service provider must be able to detect the opportunities and threats of the market on which it operates. In this context, the formulation of a realistic, coherent, and explicit strategy by the medical unit is of crucial importance in anticipating its future and reducing uncertainty in the activity.

The marketing strategy is the way an organization acts under the influence of environmental factors. In practical terms, marketing strategies outline a path following the analysis of environmental factors. The marketing policy defines its general framework of action in order to carry out its entire activity, including several strategies.

Developing the marketing policies and strategies specific to health services providing units is a complex process. Taking into account many internal and external factors, the interdependencies and conditioning links between them, as well as the favorable or unfavorable impact they can exert on the health unit, they must be analyzed in depth, interrelated, and interpreted for making strategic and firm decisions regarding the future development of the medical institution.

The core of the marketing strategy in the field of health services is represented by the quality of services. Successful organizations in the healthcare field have a clear, competitive, strategy that empowers and forces them to adapt to environmental conditions. The marketing strategy in the healthcare services field is in fact the attitude of the medical organization in relation to the marketing environment and, at the same time, its position in relation to its components.

At present, patients have so many options regarding the choice of healthcare services and providers that the only way the healthcare practices can really be distinguished is by establishing a well-differentiated, memorable, and unique proposal alongside a marketing strategy adapted to the digital era.

A valuable proposal must have the following characteristics:

— It is true
— The value offered is superior to the one of the competition
— It is important to the target public
— It is memorable and easy to remember
— It is difficult to copy by the competition.

According to a Harvard Business Review, 64% of the consumers have “genuine common values” as the main influence on their relationship with the brand (Hirsch L. The 8 Most Important Marketing Strategies for a Healthcare Practice – Part 1, November 22, 2017, retrieved from: https://hirschhealthconsulting.com/8-important-marketing-strategies-healthcare-practice-part-1/).

Today, major healthcare organizations focus on content to win the race of digital supremacy. Content marketing strategies for the healthcare field are not just about blogging and producing tangible results. Since hospitals are linked to patients and physicians, digital marketing is the way to bring this process to a completely new level.

At present, digital content helps build positive brand impressions. The use of new digital marketing strategies is essential to maximize the efficiency of marketing expenses and generate higher return rates. By applying innovative health marketing principles to reinvigorate the medical organization’s marketing initiatives, organizations will be able to better position their service offers to consumers.

For healthcare providers, the use of informative blogs or articles published on social media can be effective ways to stay relevant to patients. Moreover, infusing targeted keywords into the content can add a marketing boost.

For organizational marketing to be effective, the digital platforms in which the organization will operate on must be identified, the target public will have to be segmented correctly, and customized marketing messages will have to resonate with the audience.

**Figure 1:**
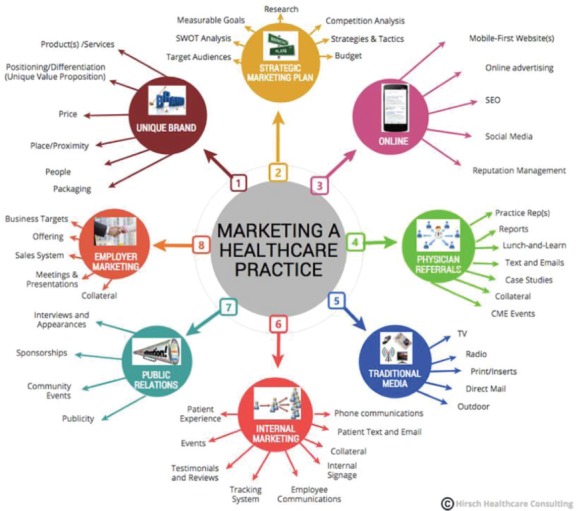
Marketing strategies for marketing a healthcare practice in the healthcare field. Source: Hirsch Healthcare Consulting, https://hirschhealthconsulting.com/8-important-marketing-strategies-healthcare-practice-part-1/.

To understand the impact of marketing strategies on the quality of healthcare services, it is important to understand today’s medical consumer who prefers to look for medical information online, where he also has a wealth of healthcare services, healthcare providers, reviews from patients who contacted the provider, etc.

With digital marketing, almost everything can be tracked and measured. Healthcare professionals and healthcare organizations no longer need to insight what works and what does not work. With the help of marketing performance information, healthcare professionals, and healthcare organizations can make an informed decision on how to improve their efforts, along with the ability to continually measure and evaluate them.

The healthcare industry has the potential to significantly increase its coverage and effectively engage consumers with digital marketing tactics.

As the marketing progress grows, organizations are moving towards more digital approaches to remain relevant to consumers. Digital marketing expenses have been the highest of all time, with healthcare companies spending over $ 2.5 billion on marketing, estimated at $ 4 billion by 2020 (Health Works Collective, Digital Marketing Dominates Healthcare Advertising, October 20, 2018, retrieved from: https://www.healthworkscollective.com/digital-marketing-dominates-healthcare-advertising/).

In this context, 44% of the marketing costs for health-related products and services are dedicated to mobile and digital platforms. TV advertising costs have dropped to less than 33% and are expected to continue to decrease, as the cost-effectiveness of placing a product or service on TV seems to no longer justify the investment.

The way consumers use the internet to find hospital units and healthcare providers evolves in favor of smart devices. With more than 80% of the patients who frequently use smartphones, to either identify or interact with physicians, it is essential to reconfigure marketing initiatives to better fit the era we live in. In addition, as Google reconfigures its search algorithms to favor mobile-friendly websites, now it is the right time to prioritize rethinking digital ads.

At the same time, the marketing mix strategy is necessary in medical organizations to ensure their success. Thus, the strategy leads to a significant impact on the medical organization, including its performance measured by patient satisfaction, the co-ordination of planned marketing efforts to address organizational performance being essential.

Therefore, the benefits of implementing marketing strategies are

— to improve the competitive advantage,
— to increase the visibility,
— to create a solid reputation among patients,
— to understand the needs and expectations of consumers,
— to understand the patients’ perceptions of the quality and results of their experience within the medical organization, offering memorable experiences to patients and, of course, building a strong, effective, dominant brand on the health services market.

